# Serum Procalcitonin in Patients With Combined Lung Cancer and Idiopathic Pulmonary Fibrosis (LC-IPF)

**DOI:** 10.7759/cureus.9507

**Published:** 2020-08-01

**Authors:** Sherif Mohamed, Azza Abdelhaffez, Nashwa Abd El-Aziz

**Affiliations:** 1 Department of Chest Diseases and Tuberculosis: Pulmonary Medicine, Faculty of Medicine, Assiut University, Assiut, EGY; 2 Department of Medical Physiology, Faculty of Medicine, Assiut University, Assiut, EGY; 3 Department of Medical Oncology, South Egypt Cancer Institute, Assiut University, Assiut, EGY

**Keywords:** combined, idiopathic pulmonary fibrosis, lung cancer, neuroendocrine, procalcitonin, prognosis, management, metastasis

## Abstract

Background

Procalcitonin (PCT) is a potential biomarker for sepsis and acts as a guide to antibiotic administration. Previous studies showed that lung cancer (LC) may increase serum PCT levels. However, no studies addressed serum PCT in patients with combined LC and idiopathic pulmonary fibrosis (IPF): LC-IPF. We aimed to evaluate the significance of serum PCT in patients with LC-IPF.

Methods

A total of 137 patients with IPF who had complete follow-up data were reviewed. They were categorized into two groups: 30 patients with LC and IPF (LC-IPF) and 82 patients with IPF only (IPF). PCT assays in the two groups were done using the enzyme-linked immunosorbent assay (ELISA) technique.

Results

Median serum PCT (IQR) was significantly higher in patients with LC-IPF in comparison to those with IPF only (0.655± 3.60 vs 0.07 ± 0.11 ng/ml, p=0.016), respectively. LC-IPF patients with neuroendocrine (NE) component, stage IV disease, and with >2 metastatic sites had a significantly higher PCT in comparison to those with non-NE, stages I-III, and <2 metastatic sites, respectively. The presence of the NE component was the only independent risk factor predictive for PCT positivity in patients with LC-IPF; OR1.8 (95% confidence interval (CI) 0.042-2.145; p = 0.042).

Conclusion

Patients with LC-IPF have higher serum PCT levels than those with IPF alone. These levels are related to the presence of NE component, advanced cancer stage, and the presence of multiple metastases. The presence of the NE component is the only independent risk factor predictive for PCT positivity in patients with LC-IPF. Further studies are warranted.

## Introduction

Idiopathic pulmonary fibrosis (IPF) is the most common of the idiopathic interstitial pneumonias and carries the worst prognosis [1]. Despite the fact that several clinicopathologic and radiographic variables have been shown to correlate with survival in patients with IPF [2], the development of lung cancer (LC) among those patients, which ranges from 4.8 to 48% [2-4], further worsens their prognosis [2,5]. Procalcitonin (PCT) is the prohormone of calcitonin and is thought to be mainly produced in the liver by macrophages (Kupffer cells) or neuroendocrine (NE) cells [6-7]. Several studies have demonstrated that PCT is a potential biomarker for sepsis and infection and a guide to antibiotic administration [8-9]. Because of its importance, PCT has been studied in patients with either IPF alone [10] or LC alone [11-13]. Beyond its role in sepsis and guidance to antibiotic administration, it was observed that PCT has a potential role in predicting cancer in non-febrile patients, and it was useful in predicting the progression of malignancy in non-febrile cancer patients [14]. Moreover, it was reported that LC may cause false positives for PCT, particularly in cases of neuroendocrine cancers or in the presence of multiple metastases [12]. To the best of our knowledge, however, no studies had evaluated serum PCT in patients with combined lung cancer and IPF (LC-IPF). Therefore, in the current study, we aimed to evaluate the possible role of serum PCT in stable IPF patients with newly diagnosed LC (LC-IPF). Those included patients who had newly diagnosed LC at the time of diagnosis of IPF and/or developed LC (biopsy-proven) during their follow-up period. We hypothesized that serum levels of PCT could be different between stable patients with IPF alone and those with combined LC-IPF.

## Materials and methods

Study design and population

**Figure 1:**
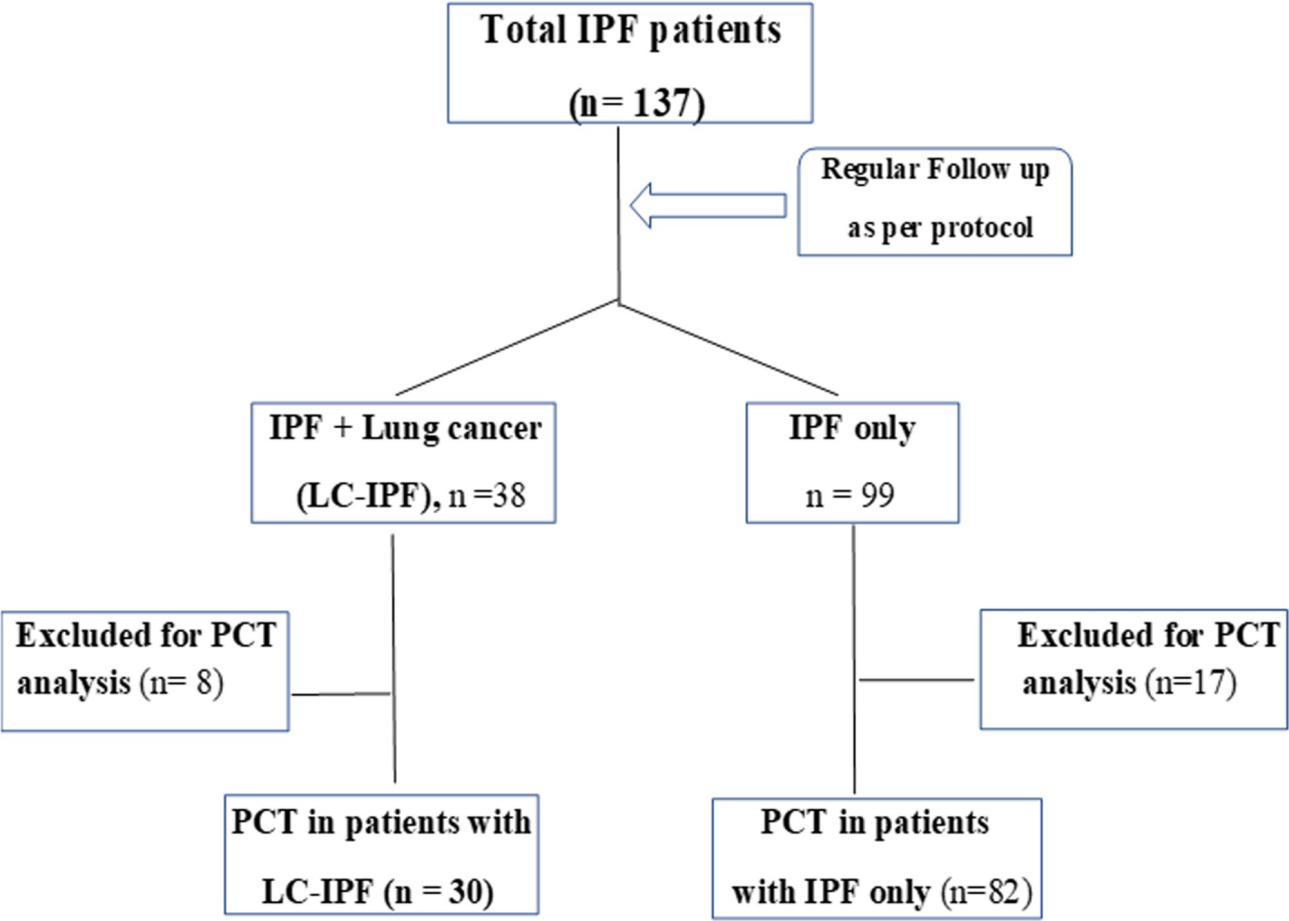
Flow chart of the study subjects

Assiut University Hospital (AUH) and South Egypt Cancer Institute (SECI) are two large tertiary hospitals serving a large number of populations at Upper Egypt. A systematic search of the patient database at the chest department of AUH revealed that 152 patients fulfilled the international guidelines on the diagnosis and management of IPF [1] during the period of August 1, 2014, to August 31, 2019. Among these 152 patients, we randomly selected 137 patients diagnosed with IPF who were then followed up regularly at our department according to a prospective protocol for the follow-up of IPF patients, as shown previously [2]. The remaining 15 patients were excluded due to incomplete follow-up data. As the study's purpose is to evaluate serum PCT only in stable IPF patients with newly diagnosed LC (LC-IPF), those 137 cases were further evaluated. Then, two groups of patients who fulfilled the enrollment criteria were chosen for comparison:

(1) The first group included IPF patients with newly diagnosed LC who had (at the time of diagnosis of IPF) or developed LC (biopsy-proven) during their follow-up period; it was named the LC-IPF group. Once diagnosed with LC, the patients were referred to the department of medical oncology at SECI for further oncologic management. Among this group, the time of the PCT assay was at LC diagnosis, and patients with the following conditions were excluded: acute exacerbation of IPF (AE-IPF), history of inflammatory disease that may modify PCT levels, clinical suspicion (including all patients with a fever greater than 38°C), or laboratory signs of bacterial or viral infection, fever of unknown origin, initiation of chemotherapy, radiation therapy, or any history of either, comorbidities that could affect the serum levels of PCT, and the use of anti-inflammatory drugs or systemic steroids. (2) The second group included those patients with IPF only (IPF group). Also, among this group, patients with the following conditions were excluded: AE-IPF, history of inflammatory disease that may modify PCT levels, clinical suspicion or laboratory signs of bacterial or viral infection, fever of unknown origin, comorbidities that could affect serum levels of PCT, and the use of anti-inflammatory drugs or systemic steroids. Then, the two groups were compared for clinical features and serum PCT levels. Figure 1 shows the flow chart of the study population.

Data collection

The medical records of all patients were reviewed to obtain data regarding age, smoking history, the method used to diagnose IPF, time of IPF diagnosis, PFT, comorbidities if any, follow-up duration, and outcomes. Pack/year value was calculated by multiplying the number of packs of cigarettes smoked per day by the number of years the person has smoked [15]. An acute exacerbation of IPF (AEIPF) was defined as acute respiratory worsening for which a cause could not be identified and meeting all criteria as proposed by the international agreement [16] and their updates [17]. Patients diagnosed as combined pulmonary fibrosis and emphysema (CPFE) were not enrolled in the study. For the LC-IPF group, all cases of LC were biopsy-proven and the time of diagnosis of LC was determined. Lung cancers were classified according to the World Health Organization (WHO) classification. The staging of LC has been established by the tumor, node, metastasis (TNM) system current at the time of diagnosis.

PCT and C-reactive protein (CRP) measurements

All serum PCT measurements were performed in the medical physiology laboratory, blinded to the clinical data. Two mL venous blood was collected once from every patient in the two groups. Sera were separated and stored at -20°C till the time of analysis. Serum PCT was measured quantitatively by the enzyme-linked immunosorbent assay (ELISA) technique using the RayBio® Human PCT ELISA kit (RayBiotech Inc., Norcross, GA) according to the manufacturer's protocol. Values were expressed in nanograms per milliliters (ng/ml). The test was considered positive when >0.10 ng/ml. Clinically relevant PCT cut-off values were determined by using those reported in published decision-making algorithms: 0.25 and 0.5 ng/ml [18-19]. Serum CRP levels were assayed by immunonephelometry used as an automated system (Vista 1500 Siemens, Munich, Germany). The test was considered positive if >5 mg/dL. As the current study includes the laboratory assessment of a biomarker, as well as all the study procedures that were neither harmful nor breaking the patients’ safety or privacy, there was no need to get approval by the local ethical committee.

Statistical analysis

Comparing the distribution of non-normally distributed variables was done using the Mann-Whitney U test in the case of two samples and the Kruskal-Wallis test for more than two samples. To identify risk factors associated with PCT positivity, a binary logistic regression model was used. If variables showed a significant difference in the univariate analysis, they were entered into a multivariate model. The odds ratio was given with the respective 95% confidence interval (CI). All tests were two-sided and a p-value of <0.05 was considered significant. Statistical analyses were carried out using the Statistical Package for the Social Sciences (SPSS) software, Version 22.0 (IBM Corp., Armonk, NY).

## Results

Patients’ characteristics

Out of 137 patients with IPF who were followed up, 38 had the diagnosis of LC-IPF. So, the prevalence of LC in IPF patients was 38/137 (27.7%). The number of patients who did not fulfill the criteria for PCT measurement was eight and 17 among patients with LC-IPF and those with IPF only, respectively. PCT was successfully measured in 30 and 82 patients with LC-IPF and IPF only, respectively, and constituted the two study groups (Figure 1).

The differences between the demographic characteristics are shown in Table 1. There were only significant differences between the two study groups with regards to gender and smoking status, p = 0.001 and p = 0.005, respectively. There were no significant differences in pack/year, medical comorbidities, and pulmonary function tests (Table 1).

**Table 1:** Comparison between demographic characteristics of LC-IPF and IPF only patients LC-IPF: lung cancer and idiopathic pulmonary fibrosis; IPF: idiopathic pulmonary fibrosis; IHD: ischemic heart disease; FVC: forced vital capacity; FEV1: forced expiratory volume in the first second; TLC: total leucocyte count; DLCO: diffusing capacity of the lungs for carbon monoxide; WBC: white blood cells; CRP: C-reactive protein; PCT: procalcitonin

	LC-IPF	IPF only	P
Subjects’ No (%)	30	82	0.001
Age, mean ± SD, y	57.3±8.35	55.0±7.54	0.591
Gender			0.001
Male, no (%)	23 (26)	65(74)	
Female, no (%)	7 (29)	17(71)	
Smoking status, no (%)			0.005
Current	22 (73.3)	61 (74.4)	
Past	7 (23.3)	15 (18.3)	
Never	1 (3.4)	6 (7.3)	
Pack/year	45.52 ± 6.27	42.78±8.22	0.107
Comorbidities, no (%)			
Diabetes mellitus	12 (40)	27 (33)	0.539
Hypertension	13 (43)	26 (32)	0.042
IHD	14 (46)	38 (46)	1.000
Pulmonary function tests (% pred)			
FVC	68.39±7.51	70.52±11.41	0.235
FEV1	72.27±8.86	76.18±9.31	0.034
TLC	70.39±10.22	72.61±9.89	0.121
DLCO	57.45±7.72	59.60±6.92	0.131
WBC (/µL)	12.200 (3500- 18.600	10.800 (3000- 19.200)	0.133
Serum CRP (median, mg/dL) (range)	33.20 (8.2-192.0)	13.15 (3.20-39.26)	0.002
Serum PCT (median, ng/ml), (range)	0.655 (0.01- 31.22)	0.070 (0.00- 3.00)	0.016

Serum PCT and CRP levels

Mean (standard deviation (SD)) serum PCT was 3.58 ± 7.08 and 0.28 ± 0.52 ng/ml in patients with LC-IPF and IPF only, respectively, while median serum PCT (IQR) was 0.655± 3.60 and 0.07 ± 0.11ng/ml, in the two groups, p=0.016, respectively. Mean (SD) serum CRP was 46.98 ± 21.19 and 14.19 ± 8.21 mg/dL, in patients with LC-IPF and IPF only, while median serum CRP (IQR) was 33.20 ± 8.78 and 13.15 ± 7.59 MG/dL, in the two groups, p=0.002, respectively (Table 1).

Patients with LC-IPF

Among the 30 patients with LC-IPF, eight (26.7%) were diagnosed as having primary LC at the same time as the IPF diagnosis. The other 22 (73.3%) patients developed LC 18.6 ± 15.8 months (median, 30 months; range, 2.8-64.4 months) after the diagnosis of IPF during the follow-up period. Squamous cell carcinoma (SCC; 40%) and adenocarcinoma (40%) were the most common histological findings in LC-IPF patients. Four (13.3%) patients had large cell carcinoma (LCC), with a neuroendocrine component in three of them. Two (6.7%) patients had small cell carcinoma. Four (13.3%) patients had early stage I lung cancer. Five (16.7%), nine (30%), and 12 (40%) patients had stage II, III, and IV, respectively. Twelve (40%) patients had metastases. Table 2 shows these details.

**Table 2:** Demographic characteristics of 30 patients with IPF-LC LC: lung cancer; IPF: idiopathic pulmonary fibrosis; NE: neuroendocrine

	LC-IPF (n)	Percent
Time of LC diagnosis		
At the time of IPF diagnosis	8	26.7
After IPF diagnosis	22	73.4
Histopathology		
Squamous cell carcinoma	12	40
Adenocarcinoma	12	40
Large cell carcinoma	4	13.3
With NE component	3	
Without NE component	1	
Small cell carcinoma	2	6.7
LC stage		
I	4	13.3
II	5	16.7
III	9	30
IV	12	40
Number of metastatic sites		
None	18	60
One	2	6.7
Two	5	16.7
Three	5	16.7
Metastasis*		
Bone	3	10
Central nervous system	3	10
Liver	3	10
Adrenal gland	1	3.3
Lung	2	6.7
Pleural	2	6.7

Serum PCT levels and patients with LC-IPF

In relation to the demographic data of patients with LC-IPF, there were significant differences between the serum levels of PCT with regards to histopathologic type, tumor stage, and the number of metastatic sites while there were no significant differences with regards to gender and CRP levels. LC-IPF patients with a neuroendocrine component, those with stage IV disease, and with more than two metastatic sites had significantly higher serum PCT levels in comparison to those with a non-endocrine component, stages I-III disease, and less than two metastatic sites, p = 0.001, p=0.014, and p=0.004, respectively. The sites of metastases included the liver, bone, and central nervous system (CNS) (10 each), lung (2), pleura (2), and adrenal glands (1) (Table 3).

**Table 3:** Relation between median serum PCT level and demographic data of patients with LC-IPF (n=30) LC: lung cancer; IPF: idiopathic pulmonary fibrosis; PCT: procalcitonin; NE: neuroendocrine

	Median serum PCT in ng/ml (±IQR)	P-value
All	0.655 ± 3.60	
Gender		0.199
Females	0.09 ± 1.29	
Males	1.33 ± 4.69	
Histopathologic type		0.001
Carcinomas with NE component	13.20±18.94	
Other histological types	0.09±1.64	
Tumor stage		0.014
Stage I to III	0.09±1.84	
Stage IV	2.14±12.65	
Number of metastatic sites		0.004
0 to 1	0.09±1.82	
2 or more	3.70±15.23	
Positive CRP		0.354
No	0.03±0.32	
Yes	1.22±2.51	

Risk of serum PCT positivity in patients with LC-IPF 

Among analyzed demographic and tumor characteristic variables, the univariate analysis showed that the presence of a neuroendocrine component is the only independent risk factor predictive for PCT positivity among patients with LC-IPF. Patients with neuroendocrine tumors had the odds ratio of 1.8 (95% confidence interval 0.042 - 2.145; p = 0.042), in comparison to those with a non-neuroendocrine component. Table 4 details these relations.

**Table 4:** Odds ratio for the risk of PCT positivity in patients with LC-IPF (n=30); univariate analysis LC: lung cancer; IPF: idiopathic pulmonary fibrosis; PCT: procalcitonin; NE: neuroendocrine; CRP: C-reactive protein

	OR (95% CI)	P-value
Gender		
Females	1	
Males	0.76 (0.511-1.185)	0.390
Histoapthologic type		
Non-NE differentiation	1	
NE differentiation	1.8 (0.042-2.145)	0.042
Tumor stage		
Stage I to III	1	
Stage IV	0.50 (0.191-1.310)	0.264
Number of metastatic sites		
0 to 1	1	
2 or more	0.42 (0.136-1.350)	0.245
CRP		
Negative	1	
Positive	0.92 (0.730-1.118)	1.000

## Discussion

To the best of our knowledge, this is the first study that addresses the value of serum procalcitonin in stable patients with combined lung cancer and idiopathic pulmonary fibrosis. In comparison to stable patients with IPF alone, patients with LC-IPF had significantly higher PCT levels (p=0.016). LC-IPF patients with a neuroendocrine component, with advanced-stage cancer, and with more than two metastatic sites had significantly higher serum PCT levels. The presence of a neuroendocrine component is the only independent risk factor predictive for PCT positivity.

This is the first study that compares the serum levels of the commonly used marker procalcitonin between patients with IPF and those with combined LC-IPF. Previous reports had addressed levels of PCT either in acute exacerbations of interstitial lung disease [10] or LC alone [11-13], with mixed results. In the current study, we had chosen well-characterized groups of stable IPF alone and LC-IPF patients who had no known factors compromising the evaluation of PCT levels (i.e., infectious, inflammatory, or therapy-induced factors). Despite the fact that IPF is associated with LC development, the common underlying mechanisms connecting both conditions are poorly understood [5]. Many theories are taken into consideration [2,5], but the exact pathogenesis is unknown; this could be reflected in the pathobiology of LC in patients with IPF and in the PCT levels in patients with LC-IPF. Despite the fact that PCT has been established as a significant biomarker of sepsis and antibiotic stewardship [7], the pathophysiology of PCT production is quite complex [6-9]. Procalcitonin is a prohormone (precursor) of calcitonin (CT). Procalcitonin is a member of the CAPA family of proteins. The biological role of calcitonin and its prohormone remains unclear. Following transcription (CALC-1 gene) and translation (calcitonin-mRNA), pre-PCT is formed. Thereafter, it is cleaved to PCT, a 116 amino-acid moiety. Further enzymatic modifications yield the 32 amino-acid hormone CT. Normally, CALC-1 gene activity is confined to the neuroendocrine cells of the thyroid and lung and very little is present in the circulation (<0.05 ng/mL) [20]. In response to inflammatory triggers (e.g. injury, burns, and infection) humoral factors are released and the complement and coagulation cascades activated. The slew of secreted factors includes acute-phase proteins (e.g., CRP), stress hormones (e.g., catecholamines), hormokines (e.g., calcitonin and its precursors, adrenomedullin and interleukin 6 (IL-6)), intracellular factors (e.g., heat shock proteins), and cytokines (immunoregulatory, pro-inflammatory, and anti-inflammatory) [7,20].

Our study observed that stable patients with LC-IPF had significantly higher PCT levels than stable patients with IPF alone. This difference is closely related to the development of LC in patients with IPF, as the majority (73.3%) of IPF patients developed LC 18.6 ± 15.8 months after the initial diagnosis of their IPF. Our finding is in agreement with many reports that observed that PCT can be elevated due to malignancy [11-14,21-22]. Ghillani et al. found increased calcitonin precursor levels as compared to healthy subjects in 17.5%, 53%, and 29% of patients with squamous cell cancer, large cell cancer, and adenocarcinoma, respectively [21]. Matzaraki and coworkers, in their study of 43 patients with solid tumors and 15 healthy controls, found that an increase in PCT in cancers that were parallel to the cancer stage, the highest levels being found in those with generalized cancer [22]. However, these two studies [21-22] included cancers of mixed origin (breast, lung, urogenital system, carcinoma of unknown origin, and others), adding to the fact that patients were undergoing chemotherapy; both these could modify PCT levels. Recently, Chaftari et al. suggested a potential role for PCT and IL-6 in predicting cancer in non-febrile patients [14]. In addition, PCT was useful in detecting the progression of cancer and predicting bacteremia or sepsis in febrile cancer patients. On the other hand, Giovanella et al., in a large series of 447 solid tumors (breast, head and neck, ovary, cervix, and NSCLC), but excluding neuroendocrine, observed that these solid carcinomas ‘‘per se’’ did not increase circulating PCT concentrations (no patient had a PCT concentration > 0.5 ng/mL), regardless of the histotype and stage of the disease [23].

In our cohort of LC-IPF patients, those with a neuroendocrine component, advanced-stage disease, and with more than two metastatic sites had significantly higher serum PCT levels in comparison to those with a non-endocrine component, stages I-III disease, and less than two metastatic sites, respectively. These findings are similar to, and supported by, the findings of previous reports. PCT is secreted ubiquitously by neuroendocrine cells located in the lung, adrenal, liver, kidney, adipose tissue, and muscles in response to sepsis [24-25]. Cate et al. had demonstrated the para-neoplastic hormonal production of calcitonin in small cell carcinomas of the lung [26]. Indeed, paraneoplastic secretion from adrenal or liver, which are common metastatic sites in LC and were seen in three (10%) of our patients, may also explain the trend for PCT positivity in metastatic diseases. Finally, the presence of neuroendocrine cells in the lung itself may also explain the risk of false-positivity [12,27]. Forty percent of our cohort had stage IV disease, and median serum PCT was significantly higher in those with stage IV in comparison to those with stages I-III; p=0.014. This finding is in agreement with that observed by Chaftari et al. [14]. They found that non-cancer patients had lower procalcitonin levels on average as compared to patients with stage I-III cancer (0.029 ng/mL vs 0.127 ng/mL, p < 0.0001) or stage IV disease (0.029 ng/mL vs 0.190 ng/mL, p<0.0001). Cancer patients who have an accelerated developmental stage have a higher mean of PCT than those in the low stages (0.190 ng/mL vs 0.127 ng/mL, p=0.004 [14]. Also, our observations are similar to those of Avrillon and her colleagues [12] who evaluated the serum PCT levels in 89 newly diagnosed LC patients. They found that PCT was positive in 42% of patients. A neuroendocrine component, having two or more metastatic sites, having pleura or liver metastasis, and being positive for CRP were all significantly associated with positive PCT in the univariate analysis. In the multivariate analysis, only the presence of a neuroendocrine component remained strongly associated with a positive PCT [12].

Despite the small number of our enrolled patients, the current study might have significant clinical implications. During regular follow-up of patients with IPF, the presence of abnormally high serum PCT in the absence of known triggers (i.e., infection, sepsis) should alert the physician to the possible presence of LC [14]. In daily clinical practice, it may be difficult for the physician to diagnose underlying cancer in the presence of pneumonia. The regression of post-pneumonia opacities may take several weeks, and such opacities may hide an LC. The incidence of LC in patients who had been hospitalized with pneumonia was significantly higher than in the general population [28]. This is expected to be more challenging in patients with LC-IPF [2], particularly those who receive chemo-or radiotherapy for their disease [2,4]. The use of sensitive biomarkers, like PCT, could be helpful in that situation. On the other hand, infections are encountered in the course of approximately one-third of LC, which may significantly impact their survival [29]. The difficulties in diagnosing infection in this group of patients are well-known. Radiological, as well as microbiological, methods can be insufficient for diagnosing infection. Again, in this dilemma, the use of PCT will be helpful especially in patients with LC-IPF, but applying the clinical thresholds for positive PCT levels of 0.25 and 0.5 ng/ml in patients with LC-IPF should be carried out with caution [12]. The current study has some limitations. First, it is retrospective, although consisting of prospectively collected data for consecutive patients admitted to our hospital. Second, the number of patients is relatively small, and the study was performed at a single center. Therefore, larger prospective studies in multi-centers are encouraged.

## Conclusions

The findings of the current study indicate that patients with combined IPF and lung cancer have higher serum procalcitonin levels than those with IPF alone. These high levels were related to the presence of a neuroendocrine component, advanced cancer stage, and the presence of multiple metastases. The presence of a neuroendocrine component is the only independent risk factor predictive for PCT positivity among patients with LC-IPF. These high PCT levels should be taken into consideration upon follow-up of IPF patients for the development of LC, as well as upon the management of infections and/or sepsis in patients with LC-IPF. Further studies are warranted.

## References

[1] Raghu G, Collard HR, Egan JJ (2011). An official ATS/ ERS/JRS/ALAT statement: idiopathic pulmonary fibrosis: evidence-based guidelines for diagnosis and management. Am J Respir Crit Care Med.

[2] Mohamed S, Bayoumi H, El-Aziz N, Mousa E, Gamal Y (2018). Prevalence, risk factors, and impact of lung cancer on outcomes of idiopathic pulmonary fibrosis: a study from the Middle East. Multidiscip Respir Med.

[3] Lee KJ, Chung MP, Kim YW (2012). Prevalence, risk factors and survival of lung cancer in the idiopathic pulmonary fibrosis. Thorac Cancer.

[4] Tomassetti S, Gurioli C, Ryu JH (2015). The impact of lung cancer on survival of idiopathic pulmonary fibrosis. Chest.

[5] Zieliński M, Sitek P, Ziora D (2018). Idiopathic pulmonary fibrosis coexisting with lung cancer. Adv Respir Med.

[6] Kretzschmar M, Kruger A, Schirrmeister W (2001). Procalcitonin following elective partial liver resection - origin from the liver?. Acta Anaesthesiol Scand.

[7] Becker KL, Nylen ES, White JC, Muller B, Snider RH Jr (2004). Clinical review 167: procalcitonin and the calcitonin gene family of peptides in inflammation, infection and sepsis: a journey from calcitonin back to its precursors. J Clin Endocrinol Metab.

[8] Schuetz P, Albrich W, Muller B (2011). Procalcitonin for diagnosis of infection and guide to antibiotic decisions: past, present and future. BMC Medicine.

[9] Yap CY, Aw TC (2014). The use of procalcitonin in clinical practice. Proc Singapore Healthc.

[10] Nagata K, Tomii K, Otsuka K (2013). Serum procalcitonin is valuable diagnostic marker in acute exacerbation of interstitial pneumonia. Respirology.

[11] Tulek B, Koylu H, Kanat F, Arslan U, Ozer F (2013). Serum C reactive protein and procalcitonin levels in non-small cell lung cancer patients. Contemp Oncol (Pozn).

[12] Avrillon V, Locatelli-Sanchez M, Folliet L (2015). Lung cancer may increase serum procalcitonin level. Infect Disord Drug Targets.

[13] Soeroso NN, Tanjung MF, Afiani D (2018). Procalcitonin level in non-small cell lung cancer patients among Indonesian population. Open Access Maced J Med Sci.

[14] Chaftari A-M, Hachem R, Reitzel R (2015). Role of procalcitonin and interleukin-6 in predicting cancer, and its progression independent of infection. PLoS One.

[15] National Cancer Institute. Definition of pack-year. https://www.cancer.gov/publications/dictionaries/cancer-terms/def/pack-year.

[16] Collard HR, Moore BB, Flaherty KR (2007). Acute exacerbations of idiopathic pulmonary fibrosis. Am J Respir Crit Care Med.

[17] Collard HR, Ryerson CJ, Corte TJ (2016). Acute exacerbation of idiopathic pulmonary fibrosis. An international working group report. Am J Respir Crit Care Med.

[18] Christ-Crain M, Stolz D, Bingisser R (2006). Procalcitonin guidance of antibiotic therapy in community acquired pneumonia: a randomized trial. Am J Respir Crit Care Med.

[19] Schuetz P, Christ-Crain M, Thomann R (2009). Effect of procalcitonin-based guidelines vs. standard guidelines on antibiotic use in lower respiratory tract infections. The ProHOSP randomized controlled trial. JAMA.

[20] Becker KL, Snider R, Nylen ES (2008). Procalcitonin assay in systemic inflammation, infection, and sepsis: clinical utility and limitations. Crit Care Med.

[21] Ghillani PP, Motté P, Troalen F (1989). Identification and measurement of calcitonin precursors in serum of patients with malignant diseases. Cancer Res.

[22] Matzaraki V, Alexandraki KI, Venetsanou K (2007). Evaluation of serum procalcitonin and interleukin-6 levels as markers of liver metastasis.. Clin Biochem.

[23] Giovanella L, Suriano S, Ricci R, Ravani P, Ceriani L (2010). Circulating procalcitonin in aseptic carcinoma patients: a specificity study with (18) F fluorodeoxyglucose positron-emission tomography/computed tomography as benchmark. Clin Chem Lab Med.

[24] Muller B, White JC, Nylen ES, Snider RH, Becker KL, Habener JF (2001). Ubiquitous expression of the calcitonin-I gene in multiple tissues in response to sepsis. J Clin Endocrinol Metab.

[25] Brunkhorst FM, Heinz U, Forycki ZF (1998). Kinetics of procalcitonin in iatrogenic sepsis. Intensive Care Med.

[26] Cate CC, Pettengill OS, Sorenson GD (1986). Biosynthesis of procalcitonin in small cell carcinoma of the lung. Cancer Res.

[27] Maruna P, Nedelnikova K, Gurlich R (2000). Physiology and genetics of procalcitonin. Physiol Res.

[28] Søyseth V, Benth JS, Stavem K (2007). The association between hospitalisation for pneumonia and the diagnosis of lung cancer. Lung Cancer.

[29] Perlin E, Bang KM, Shah A (1990). The impact of pulmonary infections on the survival of lung cancer patients. Cancer.

